# The Influence of Parents on Emotion Regulation in Middle Childhood: A Systematic Review

**DOI:** 10.3390/children9081200

**Published:** 2022-08-10

**Authors:** Karen De Raeymaecker, Monica Dhar

**Affiliations:** 1 Collaborative Antwerp Psychiatric Research Institute (CAPRI), Faculty of Medicine and Health Sciences, University of Antwerp, Universiteitsplein 1, 2610 Wilrijk, Belgium; 2 ZNA University Centre for Child & Adolescent Psychiatry Antwerp, University of Antwerp, Lindendreef 1, 2020 Antwerp, Belgium

**Keywords:** middle childhood, parents, emotion socialization, parental meta-emotion philosophy, emotional climate

## Abstract

Emotion regulation (ER) has been identified as a transdiagnostic risk factor for psychopathology, making it an ideal target for prevention and treatment. This study explores how parents can nurture the development of child ER. In April 2022, a systematic review was executed focusing on malleable factors in the parental emotion-socialization process during middle childhood. Papers in PubMed, Web of Science and Medline were screened on content-related and methodological criteria. Their methodological quality was assessed. Knowledge was assembled using a summarizing framework encompassing four factors involved in emotion socialization. Fifty papers shed light on modifiable factors at the level of parental meta-emotion philosophy, emotion-related socialization behaviors, the ER skills of parents and the emotional climate of the family. Adaptive socialization appears to be context- and child-specific, thereby taxing parents’ ER skills and their ability to put them into practice flexibly. The four changeable factors in the emotion-socialization process are highly intertwined, resulting in four possible entries for parent-directed interventions. Importantly, time should be devoted to the ER capacities of parents and their ability to attune to the situation and their child. Regarding the latter, replication studies are necessary. Recommendations for clinical interventions are provided.

## 1. Introduction

### 1.1. Background

Our lives are intersected with emotions. Difficulties in handling emotions and, more specifically, emotion regulation (ER) are prevalent in many psychological disorders (e.g., depression [1], anxiety [2], conduct problems [3], eating disorders [4] and ADHD) [5]. Consequently, as ER is considered a transdiagnostic risk factor for psychopathology [6], it has the potential to reduce or even prevent psychopathology.

Parents act as the main socializers of their children’s ER [7]. Socialization takes place through (explicit) parental emotion-related interactions and (implicit) observation of how parents themselves deal with emotion-provoking situations; these are actions that are both denoted as *emotion-related socialization behaviors* (ERSBs) [8,9]. Parents educate their children about the value of emotions, possible strategies to deal with them and their situational appropriateness (cfr., social intelligence) [8,9]. For example, when a child comes home with torn trousers after he/she fell on the playground, parents might show a worried facial expression (i.e., *emotional expressiveness*), react empathically (i.e., *reactions to the child’s emotions*), cooperatively name the emotion and think of the most optimal strategy to reduce the physiological arousal of the child (i.e., *discussion of the child’s emotions*). In the literature, strategies to encourage emotion expression and problem solving are commonly grouped as *supportive ERSBs* (e.g., emotion-focused ERSBs: “It’s OK to feel that way.”; expressive encouraging ERSBs: “If you want to cry, that’s OK.”; problem-focused ERSBs: “Maybe you can try to settle the quarrel.”), while strategies that aim to push away emotions are labelled as *unsupportive ERSBs* (e.g., punitive ERSBs: “If you don’t stop whining, you are not allowed to go horseback riding!”; minimizing ERSBs: “You are exaggerating tremendously!”; distressed ERSBs: co-rumination). These continuous learning cycles ideally result in profound knowledge to (a) recognize and label one’s own and others’ emotions (i.e., awareness), (b) appropriately express them, and (c) alter them if necessary (i.e., ER). Children who use these skills efficiently, given the situational and cultural context, will eventually reach *emotional competence* [8,10].

In these learning processes, individuals bring along their history of previous (parental) experiences [9,11]. Gottman (1996) [9] points to the formation of a *parental meta-emotion philosophy* (PMEP), based on the emotion-related socialization experiences parents had with their own parents and others (e.g., peers). As the name implies, PMEP refers to the emotions and ideas about the emotions parents have and includes the level to which parents (a) are able to internally feel and identify (low-intensity) emotions (i.e., *awareness*), (b) *accept* emotions, and (c) have a helping attitude with respect to their children’s emotions (i.e., *coaching*) [12]. PMEP impacts parents’ regulation of their own emotions and their ERSBs toward their child, thereby highlighting the possibility of intergenerational transmission through the internalization of this parental philosophy [13]. Parents with an *emotion-coaching PMEP* recognize and validate the emotions of their child, consider the experience of emotions as valuable opportunities for learning and connection, and will therefore act as coaches who help their child to understand and regulate emotions [9]. By contrast, parents with an *emotion-dismissing PMEP* use an ostrich policy to deal with emotions [9]. These parents believe that emotions are not worthy of attention. Hence, if the emotions of their child stay within relatively normal ranges, they will neglect them: children need to learn that emotions are only temporary and will generally subside. However, more extreme emotions (and certainly the negative ones) are seen as potentially dangerous. When parents notice that children experience intense emotions, they feel obliged to “chase them away” as quickly as possible, thereby depriving their child of the opportunity to collaboratively co-construct a suitable solution.

These emotion-related socialization processes occur against the *developmental background* of the child. As children mature, their ER skills become progressively internalized [14]. In middle childhood, which is defined as the period between ca. 6 and 12 years old, children progress remarkably in their emotional development [15]. Children start to realize that emotions can have an internal origin (e.g., thoughts) and are not always driven by external events [16]. They develop a sense of agency regarding self-conscious emotions (e.g., pride, guilt). Adults do not have to actively name these emotions, nor do they have to be present for children to realize when they are feeling these emotions [15]. Children in middle childhood understand that individuals can experience mixed emotions [17]. Relatedly, they discover that differences may exist between internally felt and nonverbally displayed emotions [15,18]. Additionally, they become increasingly aware of the social context that surrounds them and learn which emotion-related behaviors are not socially accepted [19]. These maturing reflections on thoughts and feelings go hand in hand with improved emotion regulation capacities. At age 10, children are able to alternate between problem-focused (action-directed) ER and emotion-focused (internally directed) ER, depending on the situation [20,21]. Consequently, children of this age increasingly understand that they can deal with feelings autonomously [7,13]. Accordingly, parents take on a more advisory role instead of a stringent directory role [15].

Another important framework for the socialization of emotions is the *emotional climate of the family* [7]. Indeed, ERSBs do not take place in isolation from general parenting practices. Gottman et al. (1996) [9] state that an emotion-coaching PMEP and the following ERSBs are embedded in a network of positive parenting practices. However, they do not coincide; sensitive parenting includes contingent reactions to the emotional needs (i.e., ERSB) and actions of the child (i.e., general parenting practice), developmentally relevant support and encouragement (i.e., general parenting practice) and the expression of positive affect (i.e., ERSB) [11]. Other commonly mentioned elements of the emotional family climate are attachment style, the frequency of arguments between parents and the extent to which positive and negative emotions are displayed within the family. ER and attachment are closely related, as the ability to recognize and resonate with the emotions of others is a prerequisite for secure relationships [22,23].

Furthermore, Eisenberg et al. (1996) [8] and Eisenberg (2020) [24] underscore the importance of *personal characteristics* such as the psychopathology, gender and temperament of parents and children as potential moderators of emotion-socialization processes. Ideally, parents are able to adjust their ERSBs to the level of physiological arousal of their child in order to create learning opportunities: children should neither be over-aroused nor under-aroused [8]. For example, children with a temperamental tendency to stay focused on negative experiences (i.e., “high negative affectivity” and “low effortful control”) are biologically more aroused [25], and consequently, have a tendency toward lower ER skills [26]. Moreover, in line with transactional models (e.g., [27]), scholars assume that emotion socialization is a bidirectional process in which parents and children influence each other in a reciprocal and progressively intertwined way [13,28,29].

### 1.2. Objectives

**Figure 1:**
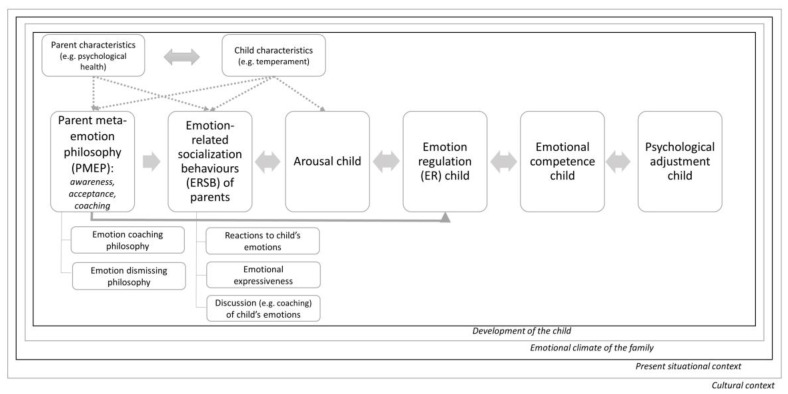
Summarizing theoretical framework.

The above-presented discussion makes clear that parents play a central role in the emotion-related development of their children. Based on the most important models of the role of parents in the development of ER [7,8,9,24], we constructed a summarizing theoretical framework (Figure 1), incorporating all the above-mentioned constructs. Accordingly, middle childhood creates new possibilities for parents to steer the emotion-related learning of their children in the right direction by intervening in a less directive and more reflection-stimulating way [15]. Due to the existing (partial) emotional dependency of children of this age [7,13], this developmental period could be extremely relevant for parental interventions. In order to empower parents in their preventive and/or correcting role with regard to psychopathology, we need to know what good parental influences encompass (given a certain developmental age). Therefore, this systematic review will focus on the question “How can parents influence the ER of their children in middle childhood in a positive way?”.

## 2. Methods

All procedures were executed in accordance with the Preferred Reporting Items for Systematic Reviews and Meta-Analyses (PRISMA) 2020 Statement [30].

### 2.1. Retrieval of Relevant Studies

In order to find an answer to our research question with the most recent evidence, we consulted published articles in the databases PubMed, Medline and Web of Science published in the last ten years (2012–2022). The initial search terms included “emotion regulation”, “emotion socialization”, “meta-emotion/metaemotion”, “parent” and “child” (and truncated forms). To reduce the large number of hits, we specified the developmental age range (“not toddler, not preschooler, not adolescent, not teen, not adult”) and indicated that we were only interested in possible changeable factors (“not illness, not medical”) and ER-related processes regarding parents (“not teachers, not peers”). Furthermore, conference materials were excluded. We limited our search to the English language. The databases were last consulted on the 12th of April 2022.

### 2.2. Selection of Relevant Studies

After reading the titles and abstracts, the selection of relevant studies was based on the following inclusion criteria: papers needed to (a) span protective and vulnerability factors concerning ER development; (b) be potentially changeable; (c) be directed at parents in relation to their children (not: studying differences between certain groups of parents or children, without specifically taking into account the parent–child relationship); (d) have a link with ER, emotion socialization or the wellbeing of children (not: school performance or social competence); and (e) refer to middle childhood (i.e., the studied age interval had to comprise at least a part of the 6–12-year age interval). Articles that predominantly focused on gender differences were excluded, as this was not the main focus of this systematic review.

Whenever each of the five inclusion criteria was met or when more information was needed to evaluate the inclusion criteria, the papers were retained for full text screening. During the second selection round, methodological quality criteria were applied. Observational studies and intervention studies needed to contain clear research questions and goals. In the case of reviews, only one of the latter had to be present. Moreover, intervention studies were only selected if they included a control group. However, in order to include the latest evidence regarding intervention studies, this quality criterion was not utilized for pilot studies. After thoughtful consideration, papers with very specific target groups (e.g., parent posttraumatic stress disorder, parents with HIV, child sexual abuse, military employment of a parent) were excluded. This was carried out in order to increase the generalizability of the findings to the “general” clinical and non-clinical population. Only studies that met both the content-related and methodological criteria were retained.

### 2.3. Quality Assessment

Based on the presence of different study types, three tools were used to assess the methodological quality of the papers. For observational studies, the Strengthening the Reporting of Observational Studies in Epidemiology (STROBE) Statement [31] was utilized. The Scale for the Assessment of Narrative Review Articles (SANRA) [32] was used to determine the quality of narrative reviews. For systematic and meta-analytic reviews, the PRISMA 2020 Statement was utilized. All three tools are checklists in which a score is given regarding the (partial) presence or absence of certain criteria. Although the checklists were not specifically designed for quality assessment, they provide a useful tool for this purpose.

In line with the SANRA, each element of the checklists was scored with 0 (not present), 1 (partially present) or 2 (present). The STROBE statement consists of 22 criteria, which means that the maximum score is 44. The SANRA has only 6 items with a maximum quality score of 12. The PRISMA 2020 Statement is, again, much longer and contains 27 criteria (maximum score: 54). All scores were added to give an overall idea of the quality of the papers. To reduce arbitrary choices, no cut-off scores were defined. Higher scores indicate better methodological quality.

Throughout the entire selection, data collection, quality assessment and synthesis, the main author of the review worked autonomously. To reduce the number of researcher-related biases, regular meetings were held with the second author, in which the inclusion criteria, the data collection and the general content of the papers were discussed.

### 2.4. Data Collection and Synthesis

Data were collected regarding (a) the study type, (b) the respondents, (c) the sample characteristics, (d) the most important study variables, (e) the used ER-related measures regarding children, (f) the ER-related measures regarding parents, and (g) the measures of ERSBs. No effect measures were reported given the large diversity of studied topics, participants and study types. The data were grouped according to the summarizing theoretical framework presented above (i.e., PMEP/mindfulness, ERSB, ER parents and the emotional climate of the family; see Figure 1). Note that the category PMEP was taken together with mindfulness as a single category since both show clear similarities (e.g., [33]). Afterward, the data were qualitatively synthesized by summarizing the main relevant conclusions per factor of the theoretical framework. Special attention was given to confirming and disconfirming evidence between different papers.

## 3. Results

### 3.1. Selection of Relevant Studies

**Figure 2:**
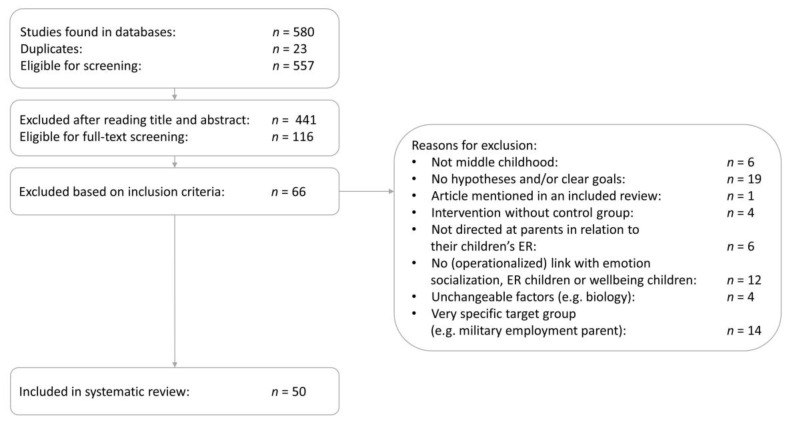
Flow chart of the screening process.

A total of 580 studies were found. After the removal of duplicates, 557 papers were eligible for screening based upon their titles and abstracts. Based on the inclusion criteria, 116 papers were retained for full-text screening. Ultimately, 50 papers were kept for further analysis (see Figure 2).

### 3.2. Study Characteristics

An overview of the incorporated studies with all their characteristics can be found in Table 1. The majority of the studies were observational and used community samples. Furthermore, we mostly found Western studies. Nine of the 50 published studies were executed with an Eastern dataset [34,35,36,37,38,39,40,41,42]. This is important, as research has shown that cultural emotion-related differences may exist. For example, minimization, an ERSB, was found to be related to worse ER of children in Western countries, but unrelated to the ER of children in a Chinese sample [43]. Nevertheless, Pinquart and Kauser (2018) [44] state that, after executing a meta-analysis on the coherence between parenting styles and psychosocial adjustment, “more ethnic and regional similarities than differences were identified” (p. 75). Advantageously, two studies [45,46] of the included study sample adopted a cross-cultural perspective in which at least two cultural backgrounds were compared. Furthermore, 22 studies overrepresented Caucasian/white/European individuals [33,45,47,48,49,50,51,52,53,54,55,56,57,58,59,60,61,62,63,64,65,66,67]. Two papers [68,69] also reported over-representing African-American individuals. Thirdly, 18 studies explicitly mentioned that conclusions were predominantly based on highly educated individuals [33,36,37,38,39,42,46,48,49,50,51,60,61,63,64,66,67,68]. These sample characteristics might limit the generalizability of the findings and should therefore be interpreted with a critical mind-set.

Moreover, while it is generally accepted that fathers (and not only mothers) play a crucial role in the ER development of children (e.g., [70,71]), 13 studies took only the influence of mothers into account [39,45,46,50,52,53,57,62,64,66,67,68,69]. Thirteen of the 24 studies that incorporated both mothers and fathers, showed a clear imbalance between the included number of mothers and fathers [33,34,35,42,47,48,49,51,59,61,72,73,74]. One study solely focused on fathers [40].

Regarding the employed methods, it is striking that the majority of the analyzed studies used subjective measures rather than objective ones, and some mentioned this as a limitation (e.g., [61,73]). On the other hand, objective observations can be seen as a snapshot that possibly does not generalize daily life [37]. Parental ER was never objectively measured, and child ER was also predominantly (23 times out of 26) gauged via subjective measures. Nevertheless, three studies combined objective observations of child ER with subjective questionnaires [37,68,69]. Likewise, more subjective measures of ERSBs (19 times out of 28) than objective ones were employed.

An analysis of the used ER measures revealed confusion about ER, emotion dysregulation and emotion lability. *Emotion regulation* refers to the ability to assess, adapt and monitor internally felt and expressed emotional reactions in order to reach one’s own goals, given the present situational context and the developmental age of the individual [75,76]. *Emotion dysregulation* is seemingly the opposite: problems with ER that create barriers to achieve those goals (e.g., impulsivity) [69,77]. Yet, even though emotions are dysregulated, efforts to control your emotions (i.e., ER) are presumably present [77]. By contrast, *emotion lability* is denoted as susceptibility to emotion-arousing stimuli (i.e., *lability*) and problems with recuperation from it, thus staying emotionally aroused (i.e., *negativity*) [78]. High emotion lability creates a bottleneck for optimal ER and may result in emotion dysregulation [69,79]. In addition, we only view the negativity aspect of the broader construct of emotion lability as an example of emotion dysregulation.

In the studied articles, the ER and emotion lability of the child were often (19 times out of 25) measured using the Emotion Regulation Checklist (ERC) [80]. The ERC consists of two subscales: the Emotion Regulation subscale and the Negativity/Lability subscale, of which the latter incorporates both aforementioned aspects of emotion lability. In the majority of the included articles, the ERC is employed in line with the concept definitions. However, four studies added both subscales to create a composite index of ER or emotion dysregulation [39,41,45,63]. The included papers will be discussed in line with the described definitions.

Relatedly, two papers on PMEP studied the externalization of PMEP, besides philosophical content [60,81]. In other words, these studies moved beyond the strict concept of PMEP and also analyzed ERSBs. This overlap is likely due to the existence of the two separate, but related, theoretical models of Eisenberg (1998) [8] and Gottman (1996) [9] in the field of parental influence on child ER. To our knowledge, this is one of the first inquiries to integrate these ideas.

The valence of the ERSB and PMEP measures in the incorporated papers was predominantly negative. When ERSBs were questioned, 20 out of 26 studies administered a questionnaire or observation of parents’ reactions to a negative, distressing child event (e.g., the Coping With Children’s Negative Emotions Scale; CCNES; [82]) [33,36,37,38,46,47,48,50,51,53,54,56,61,62,63,64,65,66,69,72]. Only one study [67] considered ERSBs regarding positive child events. Six papers studied ERSBs in response to both positive and negative events [45,52,55,58,60,81]. Likewise, studies investigating PMEP mostly administered measures that focused on parental thoughts and feelings about the negative emotions of the child [60,81]. Merely one study [52] showed interest in PMEP regarding the positive and negative emotions of the child.

### 3.3. Quality Assessment

An overview of the numeric values of the quality assessments using the STROBE Statement (maximum score: 44), the SANRA (maximum score: 12) and the PRISMA 2020 Statement (maximum score: 54) can be found in Table A1, Table A2 and Table A3 (see Appendix A). Note that for the SANRA, the criterion “appropriate presentation of data” was never scored, as statistical data were not explicitly included in the papers. This reduced the maximum score of the SANRA to 10. The total scores of the STROBE Statement ranged between 29 (ca. 66%) and 40 (ca. 91%). For the SANRA, the scores were situated between 6 and 8. However, the study of Townshend (2016) obtained a total score of 4, so it should be interpreted with caution. The only systematic review of the incorporated studies [83] received a score of 32 (ca. 59%). Even with a continuous interpretation, the latter score may seem rather low, yet some items are hard to meet when statistical analyses (cfr., meta-analysis) are not possible. In line with the latter statement, the meta-analytic review of Zimmer-Gembeck et al. (2022) [84] obtained a clearly higher score of 39.

**Table 1:** Overview of the Included Studies and their Characteristics.

Authors	Primary Study Subject	Study Type	Respondents	Sample Characteristics	Most Important Variables	ER-Related Measure Child	ER-Related Measure Parents	ERSB-Related Measure
Allen et al. (2016) [47]	EmoClim	Observational: cross-sectional	M, F and C 9–14 y	Clinical sample; predominantly Caucasian and M	ERSB, PsyAdj C, emotional reactivity C, perceived control C	CRep: adapted version of the Responses to Stress Questionnaire	N/A	Obs: worry conversation
Arellano, Gramszlo, & Woodruff-Borden (2018) [48]	ER P	Observational: case–control	M, F and C 3–12 y	Community sample; mostly European-American, married, highly educated M	ERSB, PsyAdj P	N/A	N/A	Obs during difficult or insoluble task
Bertie, Johnston, & Lill (2021) [72]	ER P	Observational: cross-sectional	Non-clinical M and F of C 3–10 y	Mostly M with a partner	ER P, ERSB, distress P	PRep: Emotion Regulation Questionnaire	N/A	PRep: CCNES
Bridgett, Burt, Edwards, & Deater-Deckard (2015) [85]	EmoClim, ER P	Narrative review	N/A	N/A	N/A	N/A	N/A	N/A
Brumariu (2015) [86]	EmoClim	Narrative review	N/A	N/A	N/A	N/A	N/A	N/A
Burgdorf, Abbott, & Szabo (2022) [49]	PMEP/MF	Observational: cross-sectional	M and F of C 3–18 y	Community sample; mostly highly educated Australians	Feasibility intervention, mindful parenting, parenting stress, ER P, PsyAdj C	N/A	PRep: CERQ (short form)	N/A
Cabecinha-Alati, Malikin, & Montreuil (2020) [50]	ER P	Observational: cross-sectional	M of C 8–12 y	Western convenience sample; mostly Caucasian, highly educated, married and middle-class	ERSB, ER P, personality P	N/A	PRep: Emotion Regulation Questionnaire and Emotion Regulation Skills Questionnaire	PRep: CCNES
Chen et al. (2021) [34]	ER P	Observational: longitudinal	M and F of C 6–13 y	Chinese; mostly M	PsyAdj C, ER P, ER C	PRep: ERC (both subscales)	PRep: DERS	N/A
Cho et al. (2022) [45]	ERSB	Observational: cross-sectional	M of C 6–7 y	Nepalese, Korean and German sample	ERSB, ER C	PRep: ERC (the two subscales were averaged)	N/A	PRep: CCNES and PRCPS
Colegrove & Havighurst (2017) [83]	ERSB	Systematic review	N/A	N/A	N/A	N/A	N/A	N/A
Craig, Goulter, Andrade, McMahon (2021) [87]	EmoClim	Observational: longitudinal	M, F and T of C from ca. 6–14 y	C with and without risk of conduct problems; black and white	PaPrac, ER C, prosocial behavior C, callous-unemotional traits C	PRep and TRep: the Social Competence Scale	N/A	N/A
Dixon-Gordon, Marsh, Balda, & McQuade (2020) [51]	ERSB	Observational: cross-sectional	M and F of C 10–12 y	Community sample; mostly white, highly educated M	ER C, ERSB, PsyAdj C and P, autonomic reactivity	PRep: ERC (both subscales)	N/A	PRep: CCNES
Dunsmore, Booker, & Ollendick (2013) [52]	PMEP/MF	Observational: cross-sectional	M and C 7–14 y with oppositional behavior	Clinical sample; predominantly European-American, married and from rural neighborhoods	PMEP (about C’s positive emotions, C’s negative emotions and the amount of emotion-related guidance C needs), ERSB, PsyAdj C	PRep: ERC (only ER subscale)	N/A	Obs: conversation about (a) a fun activity, (b) an emotion-related event (mad, sad, scared or upset), (c) an activity of last Sunday
Evans et al. (2020) [73]	EmoClim and PMEP/MF	Observational: case–control	M and F of C ca. 11 y with and without ADHD and T	Mostly M	PsyAdj C, PaPrac, mindful parenting, distress P	PRep: self-control subscale from the Social Skills Improvement System	N/A	N/A
Flujas-Contreras, Garcia-Palacios, & Gomez (2021) [74]	ER P and PMEP/MF	Intervention study	M and F of C 6 m–15 y	Mostly married M	ER P, distress P, PsyAdj C and P, mindful tendencies P	N/A	PRep: DERS	N/A
Gershy & Gray (2020) [35]	ER P and EmoClim	Observational: cross-sectional	M and F of C 6–18 y with ADHD	Eastern sample; mostly heterosexual, married, secular, with higher SES (i.c., who are able to obtain treatment)	PsyAdj C, mentalization skills P, PaPrac	N/A	PRep: DERS	N/A
Han & Shaffer (2014) [68]	EmoClim	Observational: cross-sectional	M and C 8–11 y	Community sample; mostly African-American and but racially diverse; highly educated and married	ER C, PsyAdj C, attitude toward C	PRep: ERC (only Negativity/Lability subscale) Obs: interaction with M (coded: amount of negatively shown emotions)	N/A	N/A
Han, Qian, Gao, & Dong (2015) [36]	ERSB and ER P	Observational: cross-sectional	M and F of C 7–12 y	Eastern sample; highly educated married two-parent families	ERSB, ER P	N/A	PRep: DERS	PRep: CCNES
Hong, McCormick, Deater-Deckard, Calkins, & Bell (2021) [53]	EmoClim	Observational: longitudinal	M of C 6–9 y	Predominantly highly educated and white	ERSB, ER C, household chaos	PRep: ERC (only ER subscale)	N/A	PRep: CCNES
Hurrell, Hudson, & Schniering (2015) [54]	ERSB	Observational: case–control	M and F of C 7–12 y with and without an anxiety disorder	Predominantly middle-class and Caucasian	ERSB, ER C, PsyAdj C	CRep: Emotion Expression Scale for Children and Children’s Emotion Management Scales PRep: ERC (both subscales)	N/A	PRep: CCNES
Jin, Zhang, & Han (2017) [37]	ERSB and EmoClim	Observational: cross-sectional	M and F of C 6–12 y	Eastern urban sample; mostly highly educated, married and full-time employed	ERSB, ER C, PsyAdj C, dyadic cooperation	PRep: ERC (only ER subscale) Observation: The Child Emotion Regulation Scale	N/A	PRep: CCNES
Katz, Maliken, & Stettler (2012) [12]	PMEP/MF	Narrative review	N/A	N/A	N/A	N/A	N/A	N/A
Koh & Wang (2021) [46]	ERSB	Observational: longitudinal	M and C from 4.5–7 y	European-American and Chinese-American middle-class, highly educated sample	ERSB, PsyAdj C	N/A	N/A	Obs: conversation about negative event for the C
Li, Li, Wu, & Wang (2019) [38]	ER P	Observational: cross-sectional	M and F of C 6–12 y	Eastern, highly educated, two-parent families with a high income	ERSB, ER C and P	PRep: ERC (both subscales)	PRep: DERS	PRep: CCNES
Lindsey (2020) [88]	EmoClim	Narrative review	N/A	N/A	N/A	N/A	N/A	N/A
Lobo, Lunkenheimer, Lucas-Thompson, & Seiter (2021) [81]	PMEP/MF and EmoClim	Observational: cross-sectional	Study 1: M of C 10–17 y Study 2: M, F and T of C 8-ca. 12 y	Diverse SES and ethnicity Study 1: married or cohabiting families Study 2: one-P, two-P, reconstituted families	PMEP (about negative emotions of child), ERSB, PsyAdj C and P, distress P	N/A	N/A	Obs: conversation about (a) a nice family event, (b) a difficult family event and (c) a moment in which C misbehaved
Lunkenheimer, Hollenstein, Wang, & Shields (2012) [55]	ERSB	Observational: cross-sectional	Family members of C 8–12 y and T	Mostly white; one-P, two-P, reconstituted families and other family structures	ERSB, ER C	PRep and TRep: ERC (only ER subscale)	N/A	Obs: conversation about (a) a nice family event, (b) a difficult family event and (c) a moment in which C misbehaved
Maliken & Katz (2013) [89]	ER P	Narrative review	N/A	N/A	N/A	N/A	N/A	N/A
McKee, Parent, Zachary, & Forehand (2018) [33]	PMEP/MF	Observational: cross-sectional and longitudinal	M and F of C 3–12 y	Community sample; predominantly white, highly educated, full-time employed and married M	ERSB, mindful parenting	N/A	N/A	PRep: CCNES
Miller-Slough, Dunsmore, Zeman, Sanders, & Poon (2016) [56]	ERSB	Observational: cross-sectional	M and F of C 8–12 y	Mostly Caucasian, middle-class, married two-parent families	ERSB, PsyAdj C	N/A	N/A	PRep: CCNES
Moed, Dix, Anderson, & Greene (2017) [57]	ER P	Observational: longitudinal	M and C 4 –11 y	Mostly non-Hispanic white	ER C, PsyAdj C, aversion sensitivity M	PRep: Behavior Problem Index, Positive Behavior Scale	N/A	N/A
Morelen & Suveg (2012) [58]	ERSB	Observational: cross-sectional	M and F of C 7–12 y	Predominantly white two-parent families	ERSB, ER C, PsyAdj C	PRep: ERC (only Negativity/Lability Subscale)	N/A	Obs: conversations about a moment the C was angry, happy, sad and anxious
Morelen, Shaffer, & Suveg (2016) [69]	ER P	Observational: cross-sectional	M and C 8–11 y old	Ethnically diverse sample; mostly African-American	ERSB, ER C and P	PRep: ERC (both subscales) CRep: Children’s Emotion Management Scales for Sadness, Anger and Worry Observation: Conflict Discussion task	PRep: DERS	PRep: CCNES
Morford, Cookston, & Hagan (2017) [59]	ER P	Observational: cross-sectional	M and F of C 6 m–10 y	Western and predominantly Caucasian sample; only two-parent families; mostly heterosexual, full-time employed, married M	ER P, temperament C and P	PRep: Distress Tolerance Scale	N/A	N/A
Pasalich, Waschbusch, Dadds, & Hawes (2014) [60]	PMEP/MF (study 1) and ERSB (study 2)	Observational: cross-sectional	M, F and T of C 3–12 y with callous-unemotional traits	Mostly Caucasian/Anglo-European Study 1: clinical and community sample Study 2: clinical sample; mostly two-P families and highly educated	PMEP (about their own and their C’s anger and sadness), ERSB, PsyAdj C	N/A	N/A	Obs: conversation about a past happy and sad moment
Peisch, Dale, Parent, & Burt (2020) [61]	ERSB	Observational: longitudinal	M and F of C 5–12 y	Mostly white, highly educated M	ERSB, ER C	PRep: ERC (both subscales)	N/A	PRep: the Socialization of Coping Questionnaire (negative emotions)
Perlman, Lunkenheimer, Panlilio, & Pérez-Edgar (2022) [90]	ERSB	Narrative review	N/A	N/A	N/A	N/A	N/A	N/A
Ravi et al. (2022) [62]	ERSB and EmoClim	Observational: longitudinal	M and C from 7 to 12 y	Community sample with highly novelty-sensitive children; mostly white, two-P middle- and upper-class families	Irritability level C, ERSB, parental control	N/A	N/A	PRep: CCNES
Ren, Han, Ahemaiti-jiang, & Zhang (2021) [39]	EmoClim and PMEP/MF	Observational: cross-sectional	M of C 6–12 y	Highly educated Eastern sample	ER C, PaPrac, mindful acting P, distress P	PRep: ERC (the two subscales were added)	N/A	N/A
Sanders, Turner, Metzler (2019) [91]	ER P	Narrative review	N/A	N/A	N/A	N/A	N/A	N/A
Seddon, Abdel-Baki, Feige, & Thomassin (2020) [63]	ER P	Observational: cross-sectional	M and F of C 8–12 y	Mostly white, highly educated two-P families	ERSB, ER C and P, PsyAdj C and P	PRep: ERC (the two subscales were added)	PRep: DERS	PRep: CCNES
Shaffer, Fitzgerald, Shipman, & Torres (2019) [64]	ERSB	Intervention study	M of C 5–13 y	Non-clinical sample with predominantly European American, married, highly educated M	ERSB	N/A	N/A	Obs: the Parent–Child Emotion Interaction Task: discussion of angry and sad moment
Shenaar-Golan, Yatzkar, & Yaffe (2021) [40]	EmoClim	Observational: cross-sectional	F and C 8–18 y	Israeli	Parental feelings, PsyAdj C, ER C, attachment	CRep: DERS	N/A	N/A
Thomassin, Suveg, Davis, Lavner, & Beach (2017) [65]	ER P	Observational: cross-sectional	M, F and C 7–12 y	Mostly Caucasian, two-P families	ERSB, ER C, PsyAdj C and P, interparental positive affect congruity	PRep: ERC (only Negativity/ Lability subscale)	N/A	PRep: CCNES
Townshend (2016) [92]	PMEP/MF	Narrative review	N/A	N/A	N/A	N/A	N/A	N/A
Ugarte, Liu, & Hastings (2021) [66]	ERSB	Observational: longitudinal	M of C 2–12 y	Canadian community sample; mostly middle- to upper-middle-class Caucasians	ERSB, baseline respiratory sinus arrhythmia C, PsyAdj C	N/A	N/A	PRep: Responses to Child Emotions questionnaire
Wang, Wang, Wang, & Xing (2021) [41]	EmoClim	Observational: longitudinal	M and F of C 6–10 y (Time 1) and 9–12 y (Time 2)	Eastern sample; two-P families	ER C, PaPrac, temperament C	PRep: ERC (the two subscales were added)	N/A	N/A
Yan, Schoppe-Sullivan, Wu, & Han (2021) [42]	EmoClim and PMEP/MF	Observational: cross-sectional	M and F of C 6–12 y	Eastern sample; mostly highly educated, full-time employed M of two-P families with one C	ER C and P, mindful parenting, PaPrac, coparenting quality	PRep: ERC (both subscales)	PRep: DERS	N/A
Yi, Gentzler, Ramsey, & Root (2016) [67]	ERSB	Observational: cross-sectional	M and C 7–12 y	Mostly Caucasian American highly educated M	ERSB, ER C PsyAdj C	PRep: self-control subscale of the Social Skills Improvement System-Rating Scales	N/A	PRep: Parents’ Reactions to Children’s Positive Emotions Scale
Zimmer-Gembeck, Rudolph, Kerin, & Bohadana-Brown (2022) [84]	ER P	Meta-analytic review	M and F of C 4m–18y	Mostly socioculturally diverse	ER P, ER C, PsycAdj C, PaPrac, ERSB	Mostly DERS, ERQ, or ERC	Mostly DERS or ERQ	Mostly CCNES

*Note***.** N/A = not applicable; y = years; M = mothers; F = fathers; P = parents; C = child/children; T = teachers; PMEP/MF = Parental Meta-Emotion Philosophy/mindfulness; PsyAdj = psychological adjustment; PaPrac = parenting practices (not directly emotion-focused); EmoClim = emotional climate of the family; PRep = parent report; CRep = child report; TRep = teacher report; Obs = observation; CCNES = Coping with Children’s Negative Emotions Scale; ERC = Emotion Regulation Checklist; DERS = Difficulties in Emotion Regulation Scale [93]; ERQ = Emotion Regulation Questionnaire [94]; PRCPS = Parents’ Reaction to Children’s Positive Emotions Scale [95]; CERQ = Cognitive Emotion Regulation Questionnaire [96].

### 3.4. Review of the Included Studies

#### 3.4.1. Parental Meta-Emotion Philosophy/Mindfulness

PMEP and mindfulness are considered to be related (e.g., [33]). Two of the three defining constructs of PMEP are shared with mindfulness: (a) awareness/understanding of the emotions of yourself and others and (b) acceptance of the emotions of yourself and others. Other characterizing but non-shared elements of parental mindfulness are (c) paying attention to what is happening in the present moment (during a parent–child interaction), and (d) refraining from immediate actions when confronted with emotions (i.e., non-reactivity) [33,39,92]. Both are thought to influence parenting behavior [33]. Another related concept is *mentalization capacity*, which aids in understanding others’ mental states and, therefore, their intentions. For clarity, studies addressing PMEP, mindfulness and mentalization are discussed separately.

##### PMEP

Four studies addressed an *emotion-coaching PMEP* as a factor that promotes optimal child development and even resilience [12,52,81,83]. It helps parents to read the (negative) emotions of their child correctly [83] and to consider them as an opportunity to teach their child to deal with hardship [81]. An emotion-coaching PMEP is linked with fewer internalizing [12,52,81] and externalizing symptoms [12] in children. In the study of Dunsmore et al. (2013) [52], this association was mediated by better child ER. However, this study also showed that an emotion-coaching PMEP has a flip side: better ER skills of children cohered with more *self-reported* internalizing problems and a lower *self-reported* psychological adjustment, suggesting that an emotion-coaching PMEP may stimulate the child to reflect more on their emotions, thereby increasing their awareness of internalizing symptoms. Moreover, the study suggested that an emotion-coaching PMEP is most meaningful for children with a high emotion lability, as in these children, PMEP was associated with fewer externalizing symptoms [52]. In children with low emotion lability, this effect was reversed. Again, authors refer to better emotion reflection capacities in children and parents, resulting in greater awareness of the child’s disturbing behavior, as one of the possible explanations.

In the included studies, no detrimental effect of an *emotion-dismissing PMEP* was found [81]. This is unexpected, as the papers commonly refer to other studies in which this effect was nevertheless found ([97] cited in [81]; [98] cited in [12]; [99] cited in [81]). One study came to the conclusion that children with callous-unemotional traits had mothers who were less accepting and less encouraging of the expression of anger and sadness [60].

##### Mindful Parenting

Mindful parenting is related to more supportive ERSBs and less unsupportive ERSBs, both cross-sectionally and longitudinally [33]. In addition, mindful parenting coheres with (not directly emotion-focused) parenting practices, better child ER and lower emotion lability [39,42,49,73]. Plausibly, compassion with the child (through awareness of his/her feelings), decreased parenting stress, and the absence of the urge to react explain this association [33,49,73]. The latter statement is congruent with the findings of Evans et al. (2020) [73]: mindful parenting was associated with less reactive anger from parents, which could, in turn, be related to better child ER. Finally, interventions show that mindful parenting also ameliorates parental emotion dysregulation, which presumably results in better ER modeling toward the child [49,74].

##### Mentalization Capacities

Good mentalization capacities cause parents with emotion dysregulation to abstain from negative (not directly emotion-focused) parenting practices [35]. To boost this buffering mentalization effect, two strategies can be applied: developmentally specific psycho-education [35,91] and increasing awareness of what a child is communicating (non)verbally [83].

#### 3.4.2. Emotion-Related Socialization Behaviors

Supportive ERSBs (e.g., reinterpreting) when confronted with the negative [36,37,38,46,53,54,61,81] and positive emotions of the child [67] are generally seen as advantageous for the ER development and psychological adjustment of the child. Supportive ERSBs are associated with higher child ER [36,37,38,52,53,54,61] and lower emotion lability [61]. In order to be supportive, verbal and nonverbal elements should be congruent [83]. Additionally, perseverance in emotional parent–child conversations (as opposed to quickly or abruptly ending the communication) is associated with better child ER [55]. By contrast, unsupportive ERSBs (e.g., minimization, punishment) both as a reaction to the negative [37,38,45,53,54,57,58,60,61,62,63,66,69] and positive emotions of the child [58,60,67], are considered unfavorable since they are linked to lower child ER [37,38,45,53,54,57,61,63], increased emotion lability [38,54,58,61,63,69] and lower psychological adjustment [46,62,66]. Moreover, the impact of unsupportive ERSBs on child mental health dynamically fluctuates across development; in a study by Ugarte et al. (2021) [66], children demonstrated more internalizing symptoms in the years that mothers used more unsupportive ERSBs, compared to their own average across years. Interventions directed at ERSBs show the potential to increase supportive ERSBs and reduce unsupportive ERSBs [64,69].

Some researchers find that unsupportive ERSBs are more important for child ER and psychological adjustment than supportive ERSBs. For example, Seddon et al. (2020) [63] and Yi et al. (2016) [67] found that high levels of unsupportive ERSBs, but not low levels of supportive ERSBs, were significant predictors of child internalizing symptoms. Likewise, Ravi et al. (2022) [62] reported a significant association between unsupportive ERSBs and child irritability, but did not find the inverse effect for supportive ERSBs. Indeed, the presence of supportive ERSBs does not necessarily equate to the absence of unsupportive ERSBs, and vice versa.

Four studies did not agree with the “all positive-all negative” perception regarding supportive and unsupportive ERSBs, and argued for a more nuanced image: both supportive and unsupportive ERSBs are (mal)adaptive when they are, given the context, (un)justified. This is in line with the *divergence model,* which posits that a variety of ERSBs (between or within parents) is necessary for optimal psychological adjustment as this allows for more situationally attuned ER and emotional expression ([100] cited in [56]). For example, parents who respond unsupportively to the extreme panic of the child may adaptively teach the child that his/her panic is exaggerated [56]. Regarding anger, the social convention is mostly disapproving, thereby making non-reinforcing, unsupportive ERSBs more justified. In line with this statement, fathers’ unsupportive reactions to anger were *not* related to lower child psychological adjustment, even if the child applied adaptive ER strategies [58]. Similarly, negative parental utterances during a parent–child conflict were *not* associated with lower child psychological adjustment, as long as the parental reactions were context-appropriate [57]. However, paternal unsupportive ERSBs to adaptive ER responses of the child during sad, frightening or happy personal stories were associated with lower child ER [55] and lower child psychological adjustment [58]. These unsupportive reactions are hypothesized to give rise to the perception that what you feel is socially inappropriate and, thus, e.g., embarrassing [58].

Two papers enriched the latter contextual perspective by stating that the cultural context also impacts the relationship between ERSBs and mental health/illness [45,46]. For example, parental encouragement of the self-conscious emotion pride (i.e., supportive ERSB) showed a positive, a negative and no relationship with child ER skills in an individualistic (Germany), a collectivistic (Nepal) and a merged individualistic–collectivistic (Korea) nation [45], respectively. Moreover, Chinese-American mothers’ naming and confirmation of negative emotions during dyadic reminiscing about a negative event had, apart from the culturally invariant positive effect on child psychological adjustment, an additional boosting effect on a typically Asian determinant of depression, namely social competence [46].

Additionally, three papers [51,67,90] stated that the level of emotional support the child needs is specific to the individual. Differences may arise as a consequence of the child’s age, his/her characteristics and the situation. In the study of Yi et al. (2016) [67], children with a lower ER and less self-discipline were more sensitive to the positive effect of supportive ERSBs on psychological adjustment (i.c., fewer externalizing symptoms). Dixon-Gordon et al. (2020) [51] found that children with high emotion lability and physiological arousal profited most from high unsupportive ERSBs and low supportive ERSBs. However, children with low emotion lability, high ER or lower physiological arousal showed the best outcomes when their parents were highly supportive and hardly displayed unsupportive ERSBs. Furthermore, although high dyadic synchrony (i.e., mutual attunement promoting co-regulation) and parental modeling of ERSBs are generally considered positive, overly worried restrictiveness on the part of the parent (even in situations without acute danger) reinforces anxiety in anxious children [90].

A third approach, closely connected to the second one, departs from *affective flexibility*. Research shows that, independent of the situational context, greater flexibility in inter-familial emotion language and ERSBs is linked with better child ER skills [55]. Flexibility can be a challenge, particularly if specific emotional topics (e.g., misconduct) are being addressed. By contrast, in more general and less emotional conversations (e.g., happy and difficult family moments) flexibility arises more naturally. Flexibility in expressed affect may even compensate for less situationally appropriate ERSBs. Indeed, the wide scope of displayed emotions and the opportunity to practice the alteration of affective expressions (even without explicit coaching) protects children from detrimental ER effects. However, this correcting effect was only found during happy discussions. In addition, Miller-Slough et al. (2016) [56] found that the children of parents who consistently reacted highly supportively to the negative emotions of their child suffered from more internalizing symptoms than the children of families in which (a) parents (mostly fathers) used a lot of supportive and unsupportive ERSBs or (b) responded mostly with low-supportive ERSBs (and were, thus, possibly less aware of the internalizing problems of their child).

#### 3.4.3. Parental ER

Twelve studies underscored the importance of taking the ER of parents into account when studying the relationship between ERSBs and child ER and improving it in dysregulated parents, particularly if they suffer from psychopathology [74,89,91]. Parents scoring high on ER measures more readily use supportive ERSBs ([50,84,101] cited in [35]), less unsupportive ERSBs [69] and more positive (not directly emotion-focused) parenting practices [84], which cohere with better child ER [69,84] and psychological adjustment [84]. Conversely, parents experiencing ER difficulties may cause a *“spill-over effect”* toward children [38,85], as they are unable to model a variety of strategies ([38,69,72,102] cited in [36]). Additionally, these parents use more unsupportive ERSBs [38,63,69,72,84], less supportive ERSBs [36,38] and more maladaptive (not directly emotion-focused) parenting practices (e.g., coercion) [35,42,84]. Through unsupportive ERSBs, parents possibly try to reduce the negative emotions of their child, thereby hoping to avert an escalation of contagious emotion dysregulation in both parties [69]. Ultimately, these parent–child interactions amount to low child ER skills [38,84], high emotion lability [34,38] and low psychological adjustment [84]. However, depending on the chosen assessment and the observed variable, the influence of parental ER is well-substantiated, with effect sizes of around |.30| [84].

Four papers gained a deeper understanding of the relationship between parental ER skills and child outcomes by uncovering the involved mechanisms. Arellano et al. (2018) [48] discovered that the ER skills of parents with more generalized anxiety symptoms were insufficient to meet *situation-specific emotion-coaching demands* as they were not able to discriminate which ERSBs would resolve the distress of their child. Child characteristics also reciprocally interact with lower parental ER. Children with a high negative affectivity put the *distress tolerance* (i.e., ER) of their parents under pressure, which negatively impacts emotion coaching and modeling [59]. Additionally, the presence of child externalizing symptoms hampers parental ER skills, presumably due to increased parenting stress. With time, negative parent–child transactions exacerbate externalizing symptoms [34]. Furthermore, parental *aversion sensitivity*, characterized by arousal induced by the expression of negative emotions by the child, has repercussions for the extent to which parents seize opportunities to model appropriate ER and teach the child to adaptively deal with emotions [57]. Parents’ dismissive reactions may trigger the child to react with resistance and anger, entering a vicious cycle of negative parent–child interactions ([103] cited in [57]). Since the child is left alone with difficult feelings, parental aversion-sensitivity is related to low child ER and the onset of externalizing problems at a later time [57].

Furthermore, the ER skills of one parent affect the ERSBs of the other parent (i.e., the *cross-over effect*). In the Chinese study of Han et al. (2015) [36], lower emotion dysregulation of mothers and fathers strengthened the association between supportive ERSBs of the other parent and the ER level of the child. Conversely, the dysregulation of one parent caused the child to profit less from the supportive ERSBs of the other parent. In the Chinese study of Li et al. (2019) [38], mothers’ emotion dysregulation negatively affected the number of supportive ERSBs used by fathers when confronted with the negative emotions of their child. A third study found that *interparental positive-affect congruity* (i.e., the amount to which parents share positive affect with each other) when talking about sad emotions with the child significantly reduced the amount of child emotion lability. According to Thomassin et al. (2017) [65], interparental positive-affect congruity models adaptive emotional functioning and, due to its positivity, reduces the negative feelings of the child. Psychopathology (i.c., depression of the mother) had a negative impact on the interpersonal positive affect congruity between parents, which cohered with higher child emotion lability and, eventually, more depressive symptoms in children.

#### 3.4.4. Emotional Climate of the Family

Articles discussing the emotional climate of the family focused on how parents could *indirectly* influence the ER of their child. Four different subcategories could be distinguished: (a) general parenting practices (not directly related to emotions), (b) the environment in which parenting takes place, (c) the relationship between parents and (d) the relationship between parents and children.

##### General Parenting Practices

The value of positive parenting practices and the unfavorable effect of negative parenting practices was mentioned in seven papers [35,39,41,42,62,73,87,88]. *Positive parenting practices* include, among other things, positive reinforcement if the child behaves as requested, proactive reactions if the situation threatens to get out of hand, explanation of why you, as a parent, apply certain rules, warmth and supportiveness (e.g., encouraging the child). *Negative parenting practices* are physical punishment, authoritarian control/coercion (without taking the perspective of the child into account), exaggerated anger, inconsistent demands and overall disapproval of the child.

Positive parenting practices stimulate children to practice adaptive ER strategies in emotionally safe environments [39]. It may even compensate for factors counteracting optimal mental health: in a study by Craig et al. (2021) [87], the presence of paternal warmth in childhood protected children with low ER skills against maladaptive psychological adjustment in adolescence. However, ER development is halted if emotional insecurity persists: longitudinal research shows that physical punishment [41] and authoritarian control [62] predict lower child ER/emotion lability (measured as one construct), and lower psychological adjustment years later.

Some parenting practices are not unambiguously positive: their outcome is determined in conjunction with child characteristics. Children who experience more control of negative occurrences in their lives develop better ER strategies if their parents are less directive in imposing solutions, while children who experience less control benefit most from parents who are more directive [47]. An explanation can be found in the limited possibilities of the latter group to deal with stressful experiences [47].

##### Environment in which Parenting Takes Place

The toxic influence of *stress* was a recurring theme, and was addressed in five articles [39,72,73,81,85]. Stress (e.g., a loss of control, hopelessness and panic) creates a bottleneck for optimal parental ER as it depletes the capacity to regulate oneself (strength model of self-control: [104] cited in [85,105] cited in [39]). Stressed parents frequently use suppression to deal with their own emotions, which, in turn, leads to more unsupportive ERSBs [72]. Parental stress also operates at the philosophy level: a lack of or a lower emotion-coaching PMEP and the associated ERSBs aggravate the negative impact of stress on internalizing symptoms [81]. Likewise, stress reduces the association between parental mindfulness and positive parenting practices [39], as well as children’s ER and low emotion lability ([39,73]: ER/emotion lability measured as one concept).

Additionally, a *quiet home environment* (i.e., high predictability, not overly noisy or crowded) allows for circumstances in which ER development can thrive [85]. In their narrative review, Bridgett et al. (2015) [85] refer to several studies demonstrating a relationship between household chaos and child ER. However, Hong et al. (2021) [53] found that the influence of household chaos diminished as the child became older (i.c., an association was present at age 6 but not at age 9). According to them, child ER becomes more stable and, with the rising influence of their peers, less susceptible to a chaotic home environment. Additionally, as the effect size was small, they stipulate that the effect of household chaos may be too weak to be replicated. A third study [81] did not find a significant correlation between home chaos and child psychological adjustment.

##### Relationship between Parents

The relationship between parents can indirectly alter their ERSBs and (not directly emotion-focused) parenting practices by acting through co-parenting quality, mentioned in one paper, and martial disputes, listed in three studies. Note that co-parenting and marital relationships are different. The co-parenting relationship is primarily directed at collaborative child engagement (e.g., supporting each other in the joint effort of raising a child), while the marital relationship is centered on two individuals in a romantic relationship and their commitment toward each other ([106]; cited in [65]). A high *quality of co-parenting* strengthens the association between (not directly emotion-focused) positive parenting practices and low child emotion lability [42]. Furthermore, a positive association between co-parenting quality and child ER exists [42].

In line with the family systems theory, which states that the functioning of a family is more than just the sum of its separate parts ([107] cited in [38]), *marital disputes* tend to affect the parent–child relationship [38,83,85]. For example, maternal emotion dysregulation causes marital conflict, thereby decreasing the likelihood of fathers using supportive ERSBs and, subsequently, lower child ER [38]. Evidence on this topic is conclusive [85].

##### Relationship between Parents and Children

Four articles highlighted the role of the parent–child relationship in the development of ER. ER always takes place in an *attachment* framework [86]. In middle childhood, the secure-base (i.e., encouraging new, developmentally appropriate challenges) function of parents remains. However, the safe-haven function (i.e., ER and support when something goes wrong during the faced challenges) becomes more co-regulatory. Since attachment and ER go hand in hand, securely attached children are able to effectively regulate their emotions inside and outside the attachment relationship, even when their parents are not present. According to the narrative review of Brumariu (2015) [86], evidence regarding the different forms of insecure attachment is not strong enough to draw firm conclusions about ER or ER strategies. However, a recent study showed that child anxiety symptoms in anxiously attached children were partially explained by ER difficulties in children [40].

Relatedly, the *quality of the parent–child relationship* determines whether or not the child is receptive/resistant to the supportive ERSBs of the parent. In the Chinese study of Jin et al. (2017) [37], the relationship quality determined whether or not supportive ERSBs were related to child ER. However, this moderating effect was not found for unsupportive ERSBs.

Thirdly, a positive *affective attitude* toward the child is deemed important for the ER development of the child. In the cross-sectional research of Han and Shaffer (2014) [68], criticism toward the child was related to greater child emotion lability and, therefore, more externalizing problems. Parental emotional over-involvement (i.e., extremely positive statements during a monologue describing the child; overly protective and self-scarifying acts) was correlated with less child emotion lability and fewer externalizing problems. Although emotional over-involvement is generally linked with threatened emotional autonomy, emotional over-involvement possibly boosts the feeling of warmth and connectedness with parents, thereby increasing the probability that children internalize parents’ ERSBs [68]. Given that the majority of emotional over-involvement scores were not in extreme ranges, the authors conclude that moderate emotional over-involvement (i.e., an overly positive affective attitude) is positive in middle childhood.

## 4. Discussion

### 4.1. Summary of the Results

#### 4.1.1. General Overview

With this review, we tried to shed light on the different factors though which parents can influence their children in a positive way. For parents, middle childhood is a powerful period to impact the ER development of the child. On the one hand, children in middle childhood remarkably progress in their ER development, making them more receptive to reflective and co-regulatory ER strategies [15]. On the other hand, as the social networks of children expand, their parents remain their primary source of support [108]. These two factors make parents central figures in assisting children’s advancing knowledge and their use of diverse, situation-appropriate ER strategies [20]. Moreover, in light of inspiring new interventions directed at this time period, we only selected papers that addressed changeable factors. Taking the transdiagnostic nature of ER into account, we decided to consider studies in both clinical and community groups, thereby increasing the generalizability of our results.

We used a summarizing theoretical framework, covered in the introduction, to sort the extracted knowledge from the 50 included papers. We found evidence for changeable factors at each level of the framework (Figure 1). Consequently, four possible entries can be targeted in preventive or treatment interventions: (a) PMEP/mindfulness, (b) ERSBs, (c) the ER of the parents and (d) the emotional climate of the family. The four categories were highly intertwined. A *cascade model*, as Seddon et al. (2020) [63] proposed, seems highly plausible. Briefly summarized: low parental ER coheres with an emotion-dismissing PMEP, which causes unsupportive ERSBs and few supportive ERSBs. Consequently, the child is left with maladaptive ER strategies and insufficient ER capacity to deal with the situation on his/her own [69], resulting in low ER and low psychological adjustment.

In general, we found that an emotion-coaching PMEP, a predominance of supportive ERSBs, few unsupportive ERSBs, well-regulated parents and a stable and quiet emotional climate (with regard to both interpersonal interactions and external circumstances) are beneficial for well-developed child ER. However, some studies highlighted that the ideal circumstances to beget ER are individual- and context-specific (e.g., [52,57,67]). We conclude that unsupportive ERSBs are not unambiguously negative. Moreover, a balance between adaptive and maladaptive socialization experiences increases insight into a variety of possible ER strategies and promotes flexibility in applying them, amounting to emotional competence [55]. In line with goodness-of-fit theories (e.g., [109]), supportive and unsupportive ERSBs and their corresponding PMEPs are acceptable if they “fit” with the personal and situational demands. For example, good parental emotion-coaching capacities include finding a balance between giving the child sufficient autonomy and intervening when necessary [48].

Finding the right balance between child and situational demands can be delicate. Using supportive reactions, parents implicitly communicate to their child that their ER strategies in the present situational context are in line with the unspoken expectations. This may inadvertently reinforce the response of the child [58]. Conversely, unsupportive ERSBs result in non-reinforcement. Joint consideration of the most suitable strategy to deal with emotions is categorized as a supportive (and thus reinforcing) ERSB. Hence, tension may arise between the need for the emotional guidance of the child and the necessity to act in accordance with the situation. In this case, the situational context seems to outweigh the personal interests of the child. However, this conclusion is not robust as it is only based on children with borderline traits [51]. Spinrad, Morris and Luthar (2020) [110] suggest that validating the feeling of the child, but not his/her ER strategy, may help to reconcile both conflicting interests in order to prevent negative consequences related to child ER.

Throughout the whole socialization process, parental ER skills are extremely important, and have implications for the three other factors of the framework. Parents need to control their emotional arousal in order to respond in a non-reactive way toward the emotions of the child [111]. Parents who have better ER skills are better able to find the right balance between situational and child demands, preventing an extended duration of the child’s negative feelings and associated psychological consequences [12].

Furthermore, a good interpersonal fit contributes to the ER development of the child. Both parent–child and parent–parent relationships matter. A construct that often returns with regard to both topics is the prerequisite of emotional security. Emotional security creates a safe space to practice ER skills [99]. It advances through *attunement*, a parental state that is centered on the child and his/her emotional and physiological arousal [22]. It is frequently related to attachment [23]. However, even when parental attachment is not optimal, an emotion-coaching PMEP protects children against emotion lability and emotion dysregulation [23]. Consequently, an emotion-coaching PMEP may be a good starting point for better parental attunement with the child.

Additionally, stress and, to a lesser extent, a chaotic home environment attenuate the ER skills of children. Stress puts the ER skills of parents under pressure, making it more difficult to react supportively (e.g., [73,81]). A study by Fabes, Leonard, Kupanoff, and Martin (2001) [112] complements this finding by stating that if parents are able to refrain from displaying distress toward their child, the negative influence on the child’s psychological adjustment is limited. This, again, highlights the relevance of implementing parental ER in parental emotion-coaching interventions [50,64]. Furthermore, the possible influence of a quiet home environment in *middle childhood* remains controversial (e.g., [53,81]), yet a longitudinal study by Oloye and Flouri (2021) [113], with measures in both early *and* middle childhood, points toward the ER-thwarting effect of being raised in a noisy, crowed and disorganized environment.

#### 4.1.2. Recommendations for Clinical Practice

Throughout this review, we were confronted with many explanations to contextualize the research findings. These mediating mechanisms were, to our knowledge, often not tested in new studies. Lengua et al. (2021) [111] and McKee et al. (2018) [33] emphasize that this research domain needs more controlled intervention studies in which the mediating variables are taken onto account. Generally, three explanations were given repeatedly over the scope of all the included papers: (a) emotional resources of parents to deal with distressing situations (e.g., [48,69,72]), (b) emotional security of the child (e.g., [39,53]) and (c) situational control of parents and children (e.g., [33,73]). The three explanations give insight into the sequence that would be beneficial for parental interventions to follow.

Dysregulated parents, whose *emotional resources* fall short, are unable to assist children with their ER. The threshold at which parents are no longer able to regulate themselves varies, depending, for instance, on the temperament of the parent (e.g., high negative affectivity) and the situational context (e.g., stress) [114]. Since parental ER capacities are of tremendous importance for the emotional socialization that children receive, the ER skills of parents themselves need to be addressed first. Training these skills preferably includes (a) awareness of their own emotions (cfr., PMEP), (b) acceptance of their own emotions (building on reflections of how parents view emotions and how their ideas about emotions are influenced by their own experiences with their parents; cfr., PMEP), (c) learning a range of ER strategies, (d) psycho-education with respect to factors that may threaten ER (cfr., the emotional climate of the family), and (e) ways to deal with these threatening factors. Training these skills is meaningful as studies show amelioration after intervention [115].

Next, the *emotional security* of the child should be addressed. Parents who are able to remain regulated have sufficient emotional resources to provide emotional safety to their child. Emotional security can be promoted through an emotion-coaching PMEP and by building a close connection with the child [116]. In therapy, the latter is often achieved by encouraging parents to invest time in an activity the child prefers (e.g., [117,118]). Moreover, creating an emotion-coaching PMEP involves both awareness and acceptance of the emotions of the child. Awareness and acceptance can be promoted by increasing the mentalization capacities of parents toward the child. Examples include reflection on what the child shares (non)verbally concerning his/her emotions, and developmentally appropriate psycho-education [33,73,91]. Together, an increased understanding of the child may facilitate compassion, leniency and acceptance of the child’s emotions (e.g., [33]).

In a third phase, interventions should focus on ameliorating the *situational control* of parents (and indirectly, of the children). This phase ideally involves teaching parents how they can adaptively support their child by using child- and situation-suited ERSBs. Research shows that ERSBs can be successfully ameliorated though interventions [64,69]. We expect that, through a better parent–child relationship and increased knowledge regarding child development (i.e., emotional security), it will become easier for parents to determine what these child- and situation-appropriate ERSBs are. When the child feels supported and becomes more skillful at regulating his/her emotions, his/her sense of control over the situation will also increase. In addition, from the perspective of parents, increased control over the situation may take away the urge to react immediately when confronted with the emotions of the child (e.g., take time for a deep breath). Note that this type of parental inhibition also requires parental ER (e.g., to not become overly distressed yourself), which explains why parental ER needs to be addressed first. Like ER, the need to perceive the situation as controllable is designated as a transdiagnostic factor [119], underscoring the value of this part of the intervention.

Intervention models focusing on emotional resources, emotional security and situational control are not new. For example, the neurosequential therapy model of Perry (2006) [120] denotes the series “emotional resources, emotional security and situational control” as “*Regulate, Relate, Reason*”. Perry (2009) [121] refers to a biological foundation of his model. Regulation requires diencephalic structures. In order to be able to connect with others, limbic structures are involved. Finally, reasoning necessitates the use of cortical structures. Given that these three parts of the brain are neuro-anatomically situated above one another, it follows that individuals can not relate with each other without being regulated themselves, and vice versa. Furthermore, the “Let’s Connect” parental coaching intervention, which was included in this review, addresses socio-emotional skills before emotion communication skills [64].

Awareness (situated both at the overarching metalevel and at the momentary level) plays a dominant role in all factors of the theoretical framework we have discussed (Figure 1). Parental ER requires allowing emotions, being able to recognize and label them, understanding their origin and their behavioral effects, having an overview of possible ER strategies and choosing the right one given the situation [50]. Additionally, in PMEP, (meta-)awareness is involved (e.g., an awareness of how you view emotions, and specifically, the emotions of your child) [9]. Moreover, optimal child- and situation-specific ERSBs are based on awareness of the child’s characteristics and the context. Awareness (at meta-level) plays a role in realizing the effect of parents’ socialization behaviors on the child and his/her psychological adjustment. Parents ultimately strive to nurture the abovementioned facets of awareness within their child in order to beget emotional competence.

**Figure 3:**
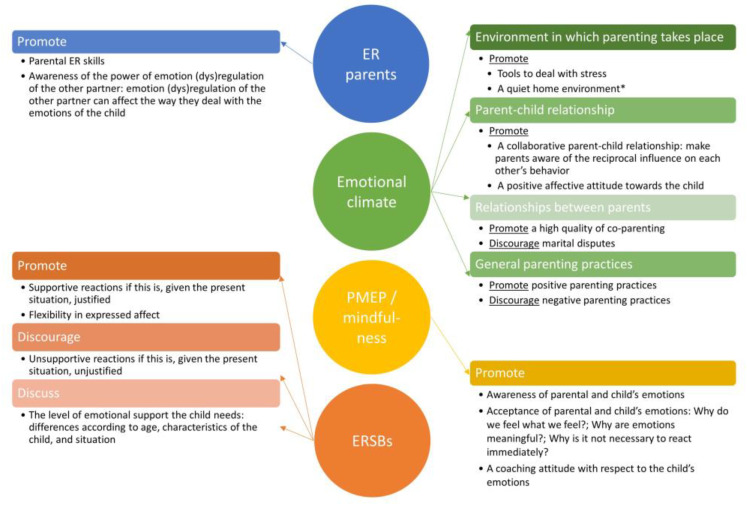
Overview of the recommendations for interventions. * Evidence for this statement is not conclusive.

Figure 3 presents an overview of the recommendations with regard to the development of new interventions. Note that the order of discussed topics slightly differs from the order used in the results section. This order was intended to be as congruent as possible with the proposed intervention sequence addressed above. Regarding the category “PMEP/mindfulness”, we assembled our review findings of the three facets PMEP, mindfulness and mentalization. Independent of the used approach, acceptance and awareness of the emotions of the child create parental compassion and leniency. Considering ERSBs, a stepwise method to increase flexibility in the expressed affect includes starting with general discussions (related to more automatic flexibility) and refraining from strongly emotional topics (e.g., child misconduct) [55]. The category “ER parents” and the subcategory “relationships between parents” of the larger category “emotional climate of the family” highlight that ideally, both parents should participate in the intervention [36,38]. However, if this is not possible due to practical reasons, it is recommended that parents should be encouraged to share the learned tips and tricks with each other.

#### 4.1.3. Limitations of the Review

Although the first and second author discussed the progress and the content of the review regularly, the quality of papers was assessed by a single researcher. Even so, this comprises a human factor that cannot be resolved by another person taking part in the process. Moreover, we consulted only English papers (of a certain quality standard), which we did not supplement with snowballing. Hence, it is possible that our selection of papers with the newest evidence-based knowledge regarding the emotion socialization of parents is not exhaustive. Relatedly, we are aware of the fact that published results are, to a certain extent, biased because of the file-drawer problem.

Secondly, in order to provide a comprehensive overview of middle childhood, we opted to include as many studies as possible incorporating children in middle childhood. Therefore, it must be noted that some studies span a broader age range than middle childhood. However, this concerns only a minority of the reviewed studies.

Thirdly, some papers were bound to methodological restrictions. As already discussed in the results section, most observational studies were cross-sectional, thereby not enabling causal conclusions. The majority of the studies suffered from the overrepresentation of certain groups (e.g., white individuals, highly educated persons, mothers). Future research will ideally be able to include a more optimal sample variation. Furthermore, subjective measures were popular. Regarding parental ERSBs, the studies mostly used the subjective measure CCNES, which has a negative valence. Studies interested in positive emotions or positive and negative emotions were scarce. However, the ER of positive emotions also provides meaningful information [56]. Lindsey (2020) [88] states that more research considering positive emotions is needed. Additionally, he argues that researchers should zoom in on the direction of ER (i.e., upregulation vs. downregulation) and the different types of ER strategies (instead of referring to the entire group of (un)supportive ERSBs).

Furthermore, evidence regarding repeatedly addressed topics (e.g., (un)supportive ERSBs) is more conclusive than that of subjects involved in one or two studies (e.g., aversion sensitivity, flexibility). The research findings of the latter category would benefit from new replication studies. Relatedly, intervention research that, besides the intervention effects, studies the involved moderators and mediators could provide more insight into how and why certain factors are important in emotion socialization [33,55].

## 5. Conclusions

As a transdiagnostic vulnerability factor involved across the continuum of subclinical to clinical psychopathology, emotion regulation has the potential to serve as a resource to prevent and remediate many problems [6]. In this systematic review, we focused on ER development in middle childhood and the role of parents in nurturing it. We tried to gain insight into factors that are feasibly changeable by parents. After a systematic search, we found mutable elements at each level of the theoretical framework guiding our search, and used this knowledge to generate recommendations for clinical practice. In line with the current need for transdiagnostic interventions [122], we hope that this review will pave the way for the development of evidence-based interventions directed at parents of children in middle childhood.

## Appendix A

**Table A1:** Quality Estimation of the Included Observational Studies Using the STROBE Statement.

Authors	1	2	3	4	5	6	7	8	9	10	11	12	13	14	15	16	17	18	19	20	21	22	*n*
Allen et al. (2016) [47]	2	2	2	2	1	1	1	2	1	0	2	2	0	1	0	1	2	2	2	2	1	2	31
Arellano et al. (2018) [48]	2	2	2	2	1	2	2	2	1	2	2	2	1	2	2	2	0	2	2	2	0	0	35
Bertie et al. (2021) [72]	2	2	2	2	1	2	2	2	2	2	2	2	0	2	1	1	2	2	2	2	1	0	36
Burgdorf et al. (2022) [49]	2	2	2	2	1	2	2	2	0	2	2	2	2	1	2	2	2	2	2	2	2	2	40
Cabecinha-Alati et al. (2020) [50]	2	2	2	2	1	2	2	2	0	1	2	2	2	2	2	1	0	2	2	2	2	2	37
Chen et al. (2021) [34]	2	2	2	2	2	2	2	2	0	0	2	2	0	1	2	2	0	2	2	2	2	2	35
Cho et al. (2022) [45]	2	2	2	2	1	1	2	2	0	0	2	1	2	2	1	1	2	2	2	2	1	2	32
Craig et al. (2021) [87]	2	2	2	2	2	1	2	2	0	0	2	2	1	2	2	2	2	2	2	2	0	2	37
Dixon-Gordon et al. (2020) [51]	2	2	2	2	1	2	2	2	0	0	2	2	2	2	2	2	2	2	2	2	2	0	37
Dunsmore et al. (2013) [52]	2	2	2	2	1	1	2	2	0	0	1	1	2	2	2	2	0	2	2	2	0	2	32
Evans et al. (2020) [73]	2	2	2	2	1	1	2	2	0	0	2	2	1	2	1	2	2	2	2	1	2	2	35
Flujas-Contreras et al. (2021) [74]	2	1	2	2	1	2	2	2	0	2	2	2	2	2	1	2	0	2	2	2	2	2	37
Gershy & Gray (2020) [35]	2	2	2	2	2	2	2	2	0	0	2	2	2	2	2	2	0	2	2	2	1	2	37
Han & Shaffer (2014) [68]	2	2	2	2	1	0	2	2	0	0	2	2	2	1	2	2	0	2	2	2	2	2	34
Han et al. (2015) [36]	2	2	2	2	1	2	2	2	0	0	2	2	2	2	2	2	0	2	2	2	2	0	35
Hong et al. (2021) [53]	2	2	2	2	1	1	2	2	1	0	2	2	2	2	2	1	2	2	2	2	2	2	38
Hurrell et al. (2015) [54]	2	2	2	2	1	1	2	2	0	0	2	2	1	2	2	2	0	2	2	2	2	0	33
Jin et al. (2017) [37]	2	2	2	2	1	0	2	2	0	0	2	2	1	2	2	2	0	2	2	2	2	0	32
Koh & Wang (2021) [46]	2	2	2	2	0	0	2	2	0	0	2	2	0	0	1	1	1	2	2	2	2	2	29
Li et al. (2019) [38]	2	2	2	2	1	0	1	2	1	0	2	2	0	2	2	2	1	2	2	2	2	2	34
Lobo et al. (2021) [81]	2	2	2	2	1	1	2	2	0	0	2	2	2	2	2	2	0	2	2	2	2	2	36
Lunkenheimer et al. (2012) [55]	2	2	2	2	1	2	2	2	0	0	2	2	2	1	2	2	2	2	2	2	2	0	36
McKee et al. (2018) [33]	2	2	2	2	1	1	1	2	0	0	2	2	1	2	1	2	2	2	2	2	2	0	33
Miller-Slough et al. (2017) [56]	2	2	2	2	1	0	2	2	0	0	0	0	2	2	2	2	0	2	2	2	2	0	29
Moed et al. (2017) [57]	1	2	2	2	2	1	2	2	1	0	2	2	1	2	2	2	2	1	2	2	1	0	34
Morelen & Suveg (2012) [58]	2	2	2	2	2	2	2	2	0	0	2	2	2	2	1	1	0	1	2	2	0	0	31
Morelen et al. (2016) [69]	2	2	2	2	1	2	2	2	0	0	2	2	2	1	2	2	0	2	2	2	0	2	34
Morford et al. (2017) [59]	2	2	2	2	1	2	2	2	0	0	2	1	2	2	2	2	0	2	2	2	2	0	34
Pasalich et al. (2014) [60]	2	2	2	2	1	1	2	2	0	0	2	2	2	1	2	2	0	2	2	2	2	2	35
Peisch et al. (2020) [61]	2	2	2	2	1	2	2	2	0	2	2	2	1	2	1	2	0	2	2	2	2	0	35
Ravi et al. (2022) [62]	1	2	2	1	2	1	1	2	0	0	2	2	1	1	2	2	2	2	2	2	2	2	35
Ren et al. (2021) [39]	2	2	2	2	1	0	1	2	0	0	2	2	0	2	2	2	0	1	2	1	2	2	30
Seddon et al. (2020) [63]	2	2	2	2	1	2	2	2	1	0	2	2	1	2	2	2	0	2	2	2	2	2	37
Shaffer et al. (2019) [64]	2	2	2	2	1	2	2	2	0	0	2	2	2	2	1	2	0	2	1	2	2	0	33
Shenaar-Golan et al. (2021) [40]	2	2	2	2	1	1	2	2	1	0	2	2	2	2	2	2	2	2	2	1	1	2	37
Thomassin et al. (2017) [65]	2	2	2	2	1	2	2	2	0	0	2	2	2	2	2	2	2	2	2	2	0	0	35
Ugarte et al. (2021) [66]	2	2	2	2	2	2	2	2	0	0	2	2	2	2	2	2	0	2	2	2	1	2	37
Wang et al. (2021) [41]	2	2	2	2	2	1	2	2	0	0	2	2	2	2	2	1	0	2	2	1	2	2	35
Yan et al. (2021) [42]	2	2	2	2	1	1	2	2	0	0	2	2	2	1	2	1	1	2	2	1	2	2	34
Yi et al. (2016) [67]	2	2	2	2	1	1	2	2	0	0	2	2	2	2	2	2	0	2	2	2	2	0	34

*Note.***Title and abstract**—1: Title and abstract; **Introduction**—2: Background/rationale, 3: Objectives; **Methods**—4: Study design, 5: Setting, 6: Participants, 7: Variables, 8: Data sources/measurement, 9: Bias, 10: Study size, 11: Quantitative variables, 12: Statistical methods; **Results**—13: Participants, 14: Descriptive data, 15: Outcome data, 16: Main results, 17: Other analyses; **Discussion**—18: Key results, 19: Limitations, 20: Interpretation, 21: Generalizability; **Other information**—22: Funding.

**Table A2:** Quality Estimation of the Included Narrative Reviews Using the SANRA.

Authors	Importance	Aims/ Questions	Description Literature Search	Referencing	Scientific Reasoning	Appropriate Presentation of Data	*n*
Bridgett et al. (2015) [85]	2	2	0	2	2	N/A	8
Brumariu (2015) [86]	2	1	0	2	2	N/A	7
Katz et al. (2012) [12]	1	1	0	2	2	N/A	6
Lindsey (2020) [88]	1	1	0	2	2	N/A	6
Maliken & Katz (2013) [89]	2	1	2	2	2	N/A	9
Perlman et al. (2022) [90]	2	1	0	2	2	N/A	7
Sanders et al. (2019) [91]	2	1	0	1	2	N/A	6
Townshend (2016) [92]	2	1	0	0	1	N/A	4

*Note*. N/A: not applicable.

**Table A3:** Quality Estimation of the Included Systematic Reviews Using the PRISMA 2020 Statement.

	1	2	3	4	5	6	7	8	9	10	11	12	13	14	15	16	17	18	19	20	21	22	23	24	25	26	27	*n*
Colegrove & Hav ighurst (2017) [83]	1	1	2	2	2	1	2	1	1	1	2	0	1	0	2	2	2	0	2	1	0	2	2	0	0	2	0	32
Zimmer-Gembeck et al. (2022) [84]	1	1	2	2	2	2	2	2	2	2	2	2	1	0	0	2	2	0	2	1	0	2	1	0	2	2	2	39

*Note.***Title**—1: Title; **Abstract**—2: Abstract; **Introduction**—3: Rationale, 4: Objectives, **Methods**—5: Eligibility criteria, 6: Information sources, 7: Search strategy, 8: Selection process, 9: Data collection process; 10, Data items, 11: Risk of bias, 12: Effect measures, 13: Synthesis methods, 14: Reporting bias assessment, 15: Certainty assessment; **Results**—16: Study selection, 17: Study characteristics, 18: Risk of bias in studies, 19: Results of individual studies, 20: Results of syntheses, 21: Reporting biases, 22: Certainty of evidence; **Discussion**—23: Discussion; **Other information**—24: Registration and protocol, 25: Support, 26: Competing interests, 27: Availability of data, code and other materials.
